# Experimental study of multiple-shot unitary channels discrimination using the IBM Q computers

**DOI:** 10.1038/s41598-025-31665-z

**Published:** 2026-02-08

**Authors:** Adam Bílek, Jan Hlisnikovský, Tomáš Bezděk, Ryszard Kukulski, Paulina Lewandowska

**Affiliations:** 1 Department of Applied Mathematics, Faculty of Electrical Engineering and Computer Science, VSB-Technical University of Ostrava, 17. Listopadu 2172/15, 70833 Ostrava, Czech Republic; 2 IT4Innovations, VSB-Technical University of Ostrava, 17. Listopadu 2172/15, 70833 Ostrava, Czech Republic; 3 Department of Mathematics, TUM School of Computation, Information and Technology, Technical University of Munich, Boltzmannstr. 3/III, 85748 Garching bei München, Germany

**Keywords:** Quantum computing, Quantum networks, Quantum channel Discrimination, Benchmarking of NISQ devices, Quantum information, Quantum simulation

## Abstract

Tasks involving black boxes appear frequently in the theory of quantum information, with quantum channel discrimination as a central example that has been deeply studied. In this work, we experimentally study the discrimination between two unitary quantum channels in the multiple-shot scenario. We challenge the theoretical results concerning the probability of correct discrimination with the results collected from experiments performed on the IBM Brisbane. Our analysis shows that neither too deep quantum circuits nor circuits that create too much entanglement are suitable for the discrimination task. We conclude that circuit architectures which minimize entanglement overhead while preserving discrimination power are significantly more resilient to hardware noise if their depth does not exceed a threshold value. Consequently, our findings necessitate a paradigm shift: for execution on noisy hardware, the theoretically suboptimal circuit is, counterintuitively, often the superior choice.

## Introduction

In the last decade, quantum computing has become a reality. Quantum algorithms of increasing complexity are being implemented on progressively more advanced quantum devices. In effect, high-quality solutions to some real-world problems are expected to arrive soon. This situation motivates the need for the certification and benchmarking of various quantum devices^1–3^. The discrimination task of quantum operators constitutes one of the certification methods and quality metrics for benchmarking quantum architectures^4,5^. The theoretical background of the quantum discrimination task has been widely developed. The primary task of discrimination involves a one-shot scenario of discrimination between quantum operators. We can imagine an unknown quantum device, a black box. The only information we have is that it performs one of two quantum operators, say $\mathscr {T}$ and $\mathscr {S}$. Our goal is two-fold. First, we want to determine the highest possible probability of correct guessing. Secondly, we need to devise an optimal strategy that maximizes the probability of success.

The problem of single-shot discrimination of quantum states was solved analytically by Helstrom a few decades ago in ^6,7^. The authors calculated the probability of correct discrimination between two quantum states using the notion of a trace norm. Next, there are many modifications of the origin problem for quantum states that were also considered^8–12^. For the discrimination tasks of quantum channels^7^, the probability of success of the discrimination can be formulated by the diamond norm^13,14^, which can be computed by semidefinite programming^13,15^. However, as the dimension of quantum channels increases, computing the diamond norm becomes inefficient^16^. In general, for quantum channels, entanglement is necessary for optimal discrimination^7^. The exception is, for example, the discrimination task between unitary channels^7,17^. Furthermore, the probability of correct discrimination between two unitary channels can be expressed in the notion of the numerical range^18^. Discrimination tasks for general quantum measurements, von Neumann measurements, or SIC POVMs were also considered in the literature^19–24^. Lastly, the theory of an indefinite causal structure is one of the attractive topics. This approach uses the notion of process matrices^25,26^, which can be seen as a generalization of quantum combs. The single-shot discrimination of the process matrices was introduced^27^.

What if we have multiple copies of quantum operators? The most general discrimination approach is known as an adaptive strategy^28^. This strategy can be described by using the term quantum combs. Here, we assume freedom in choosing the setting of quantum operations and any processing between them. In addition, numerical investigation of quantum channel discrimination showed that using indefinite causal order, we can achieve greater success with discrimination^29,30^. However, determining an optimal strategy to realize such tasks is a significant challenge in practice. This is why we often limit ourselves to parallel^31^ and sequential^32^ schemes. The parallel scheme assumes N copies of a given quantum operation distributed over N entangled systems and final measurement with no additional processing, whereas the sequential scheme assumes the implementation of N copies of the quantum operation on one system, allowing additional processing between them.

One of the first theoretical results was the study of discrimination of multipartite unitary operations^29^. The authors showed that perfect discrimination can be achieved using a parallel scheme^31^. It is also possible to design a perfect discrimination scheme of unitary channels without introducing entanglement with an auxiliary system^33^. It can be done by using the sequential approach and specific processing at the end of the circuit. On the other hand, for general quantum channels, neither the parallel nor the sequential strategy guarantees an optimal solutions^34,35^. The advantage of adaptive strategies became apparent for a task concerning entanglement-breaking channels^34^. Adaptive discrimination scenarios were also investigated in^22,32,36^. In Ref. ^37^, the authors have formulated the necessary and sufficient conditions under which quantum channels can be perfectly discriminated, while in Ref. ^22^, the authors have formulated conditions for the perfect discrimination of two measurements. Furthermore, in^38^, the authors have shown that any possible adaptive method does not offer any advantage over the parallel scheme for von Neumann measurements.

One of the first experimental results for the parallel and sequential discrimination scheme of unitary channels was presented in Ref. ^39^. In the era of NISQ devices, we need to take into account certain limitations. Due to the existing decoherence, sequential schemes could not be practical for larger numbers of copies. At the same time, parallel schemes for NISQ architectures are also not possible to implement because of the limited number of qubits. Perhaps intermediate schemes, called sequentially-paralleled, may overcome both obstacles. This scheme assumes that we have $N = w\cdot d$ copies of a quantum operator, where *w* and *d* are natural numbers. The sequentially-paralleled scheme of width *w* and depth *d* consists of *d* applications of *w* copies of the quantum operator applied simultaneously to the quantum state. The initial state $\rho$ evolves through these layers, combining parallelism within each layer and a sequential structure across layers. In general, one could consider incorporating additional intermediate processing operations between layers.

This raises the critical question of whether simpler, theoretically suboptimal circuits might prove more effective in practice. To address this, we utilize a simulator calibrated to the specific error profile of the IBM Brisbane device, providing a high-fidelity environment to test and compare the resilience of different strategies.

In this work, we present a comprehensive study of various scenarios of multiple-shot discrimination of quantum unitary channels. We consider parallel, sequential, and sequentially-paralleled quantum networks for discrimination between two qubit unitary channels. We challenge the theoretical results concerning the probability of correct discrimination with the results collected from experiments performed on the IBM Brisbane. Based on several examples, our analysis shows that neither excessively deep circuits nor those creating too much entanglement are suitable for the discrimination task.

### Paper organization

This paper is organized as follows. **Section Mathematical Preliminaries** (page 2) recalls the notation and mathematical tools necessary for our research. We then formalize channel discrimination in single- and multiple-shot settings, including perfect-discrimination criteria as well as corresponding statistical methods for analysis. At page 4, we present **Experiment 1** of discrimination with no processing between $\Phi _{\mathrm{1\hspace{-0.9mm}l}}$ and $\Phi _{\textrm{RZ}(\phi )}$: methodology (discriminator and two measurement schemes), device-aware transpilation (CNOT/ECR and mapping), and hardware results on IBM Brisbane. At page 8, we present **Experiment 2** of discrimination with processing between $U=\sqrt{X}\,\textrm{RZ}(-\pi /2N)\,\sqrt{X}$ and $V=\sqrt{X}\,\textrm{RZ}(\pi /2N)\,\sqrt{X}$, including hardware results on IBM Brisbane. **Section Noise Modeling and Ablation Analysis** (page 10) presents an analytic depolarizing model and calibrated Aer ablations (single-/two-qubit noise, $T_1/T_2$, readout, and a small coherent $\textrm{RZ}$ drift), and relates these to the observed hardware trends. Finally, the **conclusions** are presented in page 11.

## Mathematical preliminaries

Let $\mathscr {X}$ be a complex Euclidean space. Then L($\mathscr {X}$) denotes the collection of all linear mappings of the form $A: \mathscr {X} \rightarrow \mathscr {X}$. An operator $X\in \text {L}(\mathscr {X})$ is positive semi-definite if $\left\langle x\right| X\left| x\right\rangle \ge 0$ for all $\left| x\right\rangle \in \mathscr {X}$. The set of all such operators is written as $\text {Pos} (\mathscr {X})$. By $\Omega (\mathscr {X})$ we denote the set of quantum states $\rho$ $\in \text {Pos}(\mathscr {X})$ such that $\text {Tr}(\rho )=1$. Let $X\in \text {L}(\mathscr {X})$. We denote by $\text {spec(X)}$ the set of all eigenvalues of *X*.

The set of all linear mappings from L($\mathscr {X}$) to L($\mathscr {Y}$), $\Phi :\text {L}(\mathscr {X}) \rightarrow \text {L}(\mathscr {Y})$, will be denoted as T($\mathscr {X}$,$\mathscr {Y}$). The tensor product of linear maps (or operators) $\Phi$ and $\Psi$ will be denoted as $\Phi \otimes \Psi$. A linear map $\Phi \in \text {T}(\mathscr {X}$,$\mathscr {Y})$ is positive if it holds that $\Phi (P) \in \text {Pos}(\mathscr {Y})$ for all $P \in \text {Pos}(\mathscr {X})$, whereas $\Phi$ is a completely positive map (CP) if $\Phi \otimes \mathrm{1\hspace{-0.9mm}l}_{\text {L}(\mathscr {Z})}$ is a positive map for every complex Euclidean space $\mathscr {Z}$. We say $\Phi$ is trace-preserving (TP) if it holds that Tr($\Phi (X)$) = Tr(*X*) for all $X \in \text {L}(\mathscr {X})$. A linear map $\Phi \in \text {T}(\mathscr {X},\mathscr {Y})$, which is a CPTP map, is called a quantum channel. The collection of all quantum channels is denoted as $\textrm{C}(\mathscr {X},\mathscr {Y})$ (with a shorthand $\textrm{C}(\mathscr {X}) :=\textrm{C}(\mathscr {X},\mathscr {X})$). Next, we distinguish a special subset of quantum channels known as unitary channels, $\Phi _U \in \textrm{C}(\mathscr {X})$, defined as $\Phi _U(X) = UXU^{\dagger }$ where $U \in \text {L}(\mathscr {X})$ is the unitary matrix.

A Positive Operator-Valued Measure (POVM) $\mathscr {P}$ is a collection of operators, the so-called effects $\{E_0, \ldots , E_n\} \subset \text {Pos}(\mathscr {X})$ with the property of $\sum _{i=0}^{n} E_i = \mathrm{1\hspace{-0.9mm}l}$. According to the Born rule for a given quantum state $\rho$ the probability of obtaining the result $E_i$ is given by $\text {Tr}(E_i\rho )$.

A useful tool for studying the discrimination of unitary channels is the concept of the numerical range of an operator. For $X \in \text {L}(\mathscr {X})$ we define the numerical range of *X* as the set

1 $$\begin{aligned} \text {W}(X) :=\{ \left\langle x\right| X\left| x\right\rangle :\left| x\right\rangle \in \mathscr {X}, \langle x | x \rangle = 1\}. \end{aligned}$$

The Hausdorff-Töplitz Theorem^40,41^ states that W(*X*) is a convex set. If *X* is normal, the numerical range is a convex hull of its eigenvalues. For unitary matrices *U* we define the arc function $\theta (U)$ as the length of the smallest arc on the unit circle that contains all the eigenvalues of the unitary operator *U*. Mathematically, this can be expressed as

2 $$\begin{aligned} {\begin{matrix} \theta (U) :=& \min \Bigl \{ \Delta \in [0,2\pi ) \, :\, \exists \, \alpha \in [0,2\pi ) \text { such that } \\ & \text {spec}(U) \subset \{e^{i\theta } : \theta \in [\alpha , \alpha +\Delta ]\} \Bigr \}. \end{matrix}} \end{aligned}$$

Lastly, let us introduce the diamond norm in the space $\text {T}(\mathscr {X},\mathscr {Y})$. For $\Phi \in \text {T}(\mathscr {X},\mathscr {Y})$ it is defined as

3 $$\begin{aligned} \Vert \Phi \Vert _{\diamond } = \left\| \Phi \otimes \mathrm{1\hspace{-0.9mm}l}_{\text {L}(X)} \right\| _1, \end{aligned}$$

where $\Vert \Phi \Vert _1 = \max \{ \Vert \Phi (X) \Vert _1: X \in \text {L}(\mathscr {X}), \Vert X \Vert _1 \le 1 \}$ and $\Vert Y\Vert _1$ is Schatten 1-norm of $Y \in \text {L}(\mathscr {Y})$.

## Discrimination of quantum channels

We will consider the following scenario of quantum channel discrimination. Suppose that we have a classical description of two quantum channels $\Phi _0, \Phi _1 \in \textrm{C}(\mathscr {X}, \mathscr {Y})$ and a black box implementing an unknown channel $\Phi$, which either is $\Phi _0$ or $\Phi _1$. We would like to determine whether $\Phi = \Phi _0$ or $\Phi = \Phi _1$ have been hidden in the black box. To reveal the value of $\Phi$, we construct a quantum experiment consisting of an initial state $\rho \in \Omega (\mathscr {X} \otimes \mathscr {Z})$ and a binary measurement $\{E_0, E_1\} \subset \text {Pos}(\mathscr {Y} \otimes \mathscr {Z})$. The channel $\Phi \otimes \mathrm{1\hspace{-0.9mm}l}_{\text {L}(\mathscr {Z})}$ is applied to $\rho$ and then the output state $(\Phi \otimes \mathrm{1\hspace{-0.9mm}l}_{\text {L}(\mathscr {Z})})(\rho )$ is measured by $\{E_0, E_1\}$. The measurement label defines our guess about the hidden value of $\Phi$ (the label 0 associated with the effect $E_0$ indicates $\Phi = \Phi _0$). In such a setup, the probability of successful channel discrimination $p_{\text {succ}}$ is given by

4 $$\begin{aligned} p_{\text {succ}} = \frac{1}{2} \text {Tr}(E_0(\Phi _0 \otimes \mathrm{1\hspace{-0.9mm}l}_{\text {L}(\mathscr {Z})})(\rho )) + \frac{1}{2} \text {Tr}(E_1(\Phi _1 \otimes \mathrm{1\hspace{-0.9mm}l}_{\text {L}(\mathscr {Z})})(\rho )). \end{aligned}$$

The goal of our task is to construct $\rho$ and $\{E_0, E_1\}$ that maximize $p_{\text {succ}}$. The auxiliary system $\mathscr {Z}$ is of arbitrary size and provides a resource to increase the probability of success. From the Holevo-Helstrom theorem^6,42^ we know the probability of successful channel discrimination can be expressed in terms of the diamond norm

5 $$\begin{aligned} p_{\text {succ}} = \frac{1}{2} + \frac{1}{4} \left\| \Phi _0 - \Phi _1 \right\| _\diamond . \end{aligned}$$

### Statistical probability of discrimination

We report the probability of successful discrimination $p_{\textrm{succ}}=\frac{\#\text {correct}}{\text {shots}}$ for each circuit–backend pair under equal priors. Ambiguous outcomes (equal distance to both hypotheses) were resolved by a fair coin flip, as stated in the figure captions, so every run yielded a definite label.

To estimate confidence intervals for the probability of success in experiments, we use the method of Clopper and Pearson ^43^ with a 0.99 confidence level. Due to the large number of shots per discrimination task, the confidence intervals are very narrow and centered around the estimated $p_{\text {succ}}$ values.

### Single-shot discrimination of quantum unitary channels

It is worth emphasizing at this point that there is no need to use an auxiliary system $\mathscr {Z}$ for the discrimination of quantum unitary channels ^30^. Considering the task of single-shot discrimination between unitary channels $\Phi _U,\Phi _V$, the diamond norm between such channels can be expressed using the notion of the numerical range as^7,44^

6 $$\begin{aligned} ||\Phi _{U}-\Phi _{V}||_{\diamond }=2\sqrt{1-\nu ^{2}}, \end{aligned}$$

where $\nu = \min _{w \in W(V^\dagger U)} |w|$. From the above proposition, it follows that unitary channels $\Phi _U$ and $\Phi _V$ are perfectly distinguishable if and only if $0 \in W(V^\dagger U)$. The above can also be formulated as there exists a density matrix $\sigma$ such that $\textrm{tr} (V^\dagger U \sigma ) = 0$.

Using the results of^33,45,46^ we can also calculate $\nu$ using the arc function $\theta (V^\dagger U)$ defined in Eq. (2), determining the condition for perfect discrimination between *U* and *V* in the single-shot scenario. If we define $\theta :=\theta (V^\dagger U) \le \pi$ is the minimal covering arc, then $\nu =\cos (\theta /2)$ and therefore $\Vert \Phi _U-\Phi _V\Vert _\diamond =2\sin (\theta /2)$. Furthermore, we achieve perfect discrimination for unitary channels if and only if

7 $$\begin{aligned} \theta (V^\dagger U) \ge \pi . \end{aligned}$$

### Multiple-shot discrimination of unitary channels

When multiple uses of the unitary channel are available, perfect discrimination may be achieved even if the single-shot condition $\theta (V^\dagger U) \ge \pi$ does not hold^45^. The theoretically best strategy in that case is a parallel scheme which consists of *N* copies of the unitary *U* applied simultaneously to *N* quantum registers. In that case, the problem is to discriminate identity $U^{\otimes N}$ and unitary matrix $V^{\otimes N}$. Since the arc function satisfies the scaling relation $\theta ((V^{\otimes N})^\dagger U^{\otimes N}) = N \theta (V^\dagger U)$, for $N\theta (V^\dagger U)<2 \pi$, then the condition for perfect discrimination is $N \theta (V^\dagger U) \ge \pi$^33^. This leads to the condition on the minimum number of copies required for perfect discrimination is given by

8 $$\begin{aligned} N \ge \bigg\lceil \frac{\pi }{\theta (V^\dagger U)} \bigg\rceil . \end{aligned}$$

Thus, even when $\theta (V^\dagger U) < \pi$, as long as $\theta (V^\dagger U) > 0$, perfect discrimination can still be achieved by using a sufficient number of copies of *U* and *V*.

### Experimental set-up

A wide range of multi-shot discrimination strategies has been explored in the literature. In this work, we restrict our attention to three representative classes: parallel schemes, sequential schemes, and rectangular hybrid schemes that interpolate between these two. Experimental comparison of the performance of these strategies constitutes the main contribution of our work. Our focus is on the discrimination of qubit unitary channels *U* and *V*. For simplicity we will consider only the cases that satisfy

9 $$\begin{aligned} \theta (V^\dagger U) = \frac{\pi }{N}. \end{aligned}$$

Let $N = wd$ for $w,d \in \mathbb {N}$. In our set-up, *N* copies of the black box $\Phi$ are arranged in a rectangular layout over *w* qubits with circuit depth *d*. On each qubit, the unknown operation is applied *d* times, namely, we have

10 $$\begin{aligned} \Phi ^{\otimes w} \Phi _{X_{d-1}} \Phi ^{\otimes w} \cdots \Phi _{X_1} \Phi ^{\otimes w}, \end{aligned}$$

where $X_1,\ldots ,X_{d-1}$ are arbitrary unitary matrices defined on *w* qubits, which are responsible for additional processing. In special cases, when $w=N$ and $d=1$, we obtain the parallel scheme $\Phi ^{\otimes N}$ (no processing is needed) and when $w=1$ and $d=N$, we obtain the sequential scheme $\Phi \Phi _{X_{d-1}}\cdots \Phi _{X_1}\Phi$.

11 $$\begin{aligned} \theta (((V^\dagger U)^d)^{\otimes w} )=w\theta ((V^\dagger U)^d )=wd\theta (V^\dagger U )=N \frac{\pi }{N}=\pi . \end{aligned}$$

The only remaining question is how to find optimal $\rho$ and $\{E_0, E_1\}$. The precise form of these variables depends on *U*, *V*,^7,13,44^, and will be calculated later for each pair of unitary channels considered.

## Experiment 1—Discrimination of unitary channels on IBM Brisbane without processing

This section is divided into four main parts. At the beginning, we will discuss in detail the components of discrimination schemes and later on their decompositions into native gates. Next, we will talk about transpilation approaches, and finally we will present the results obtained from the IBM Quantum device. All experiments were executed on the IBM Brisbane. Throughout, we use IBM Quantum for the platform and IBM Brisbane for the device.

### Methodology and setup

In this example, we will distinguish between identity $\Phi _{\mathrm{1\hspace{-0.9mm}l}}$ and $\Phi _{\text {RZ}(\phi )}$ for RZ$$(\phi ) = \begin{pmatrix} e^{-i\frac{\phi }{2}} & 0\\ 0 & e^{i\frac{\phi }{2}} \end{pmatrix}$$ without processing between the particular application of the unitary channel ($X_i = \mathrm{1\hspace{-0.9mm}l}$ for each $i=1,\ldots ,d$). Let $N = wd$ for $w,d \in \mathbb {N}$. In our setup *N* copies of the black box $\Phi$ can be composed in the rectangular shape spread on *w* qubits with the depth of the circuit *d*. On each qubit the unknown operation is composed *d* times, namely, we have

12 $$\begin{aligned} \underbrace{\Phi ^d \otimes \cdots \otimes \Phi ^d}_{w}. \end{aligned}$$

To determine a discriminator $\left| \psi \right\rangle$, we need to find a unit vector satisfying

13 $$\begin{aligned} \left\langle \psi \right| \text {RZ}(d \phi )^{\otimes w} \left| \psi \right\rangle = 0. \end{aligned}$$

The most distant pair of eigenvalues of $\text {RZ}(d \phi )^{\otimes w}$ are $-i$ and *i*, corresponding to eigenvectors $\left| 0\cdots 0\right\rangle$ and $\left| 1\cdots 1\right\rangle$, respectively. Hence, we can show that

14 $$\begin{aligned} \left| \psi \right\rangle = \frac{1}{\sqrt{2}}\left( \left| 0\cdots 0\right\rangle + \lambda \left| 1\cdots 1\right\rangle \right) \in \mathbb {C}^{2^w}, \end{aligned}$$

for any unit number $\lambda \in \mathbb {C}$. If $\Phi = \Phi _\mathrm{1\hspace{-0.9mm}l}$, the output state is equal to $\left| \psi _0\right\rangle = \left| \psi \right\rangle$. Otherwise, if $\Phi = \Phi _{\text {RZ}(\phi )}$, then the output states are equal to $\left| \psi _1\right\rangle = \text {RZ}(d \phi )^{\otimes w}\left| \psi \right\rangle = \frac{-i}{\sqrt{2}}\left( \left| 0\cdots 0\right\rangle -\lambda \left| 1\cdots 1\right\rangle \right)$. As $\left| \psi _0\right\rangle$ and $\left| \psi _1\right\rangle$ are orthogonal, we can find $E_0 \ge \left| \psi _0\right\rangle \left\langle \psi _0\right|$ and $E_1 \ge \left| \psi _1\right\rangle \left\langle \psi _1\right|$ (in particular $E_0 = \left| \psi _0\right\rangle \left\langle \psi _0\right|$ and $E_1 = \mathrm{1\hspace{-0.9mm}l}- E_0$), which guarantees $p_{\text {succ}} = \frac{1}{2} \text {Tr}(E_0(\Phi _{\mathrm{1\hspace{-0.9mm}l}^{\otimes w}})(\left| \psi \right\rangle \left\langle \psi \right| )) + \frac{1}{2} \text {Tr}(E_1(\Phi _{\text {RZ}(d \phi )^{\otimes w}})(\left| \psi \right\rangle \left\langle \psi \right| )) = \frac{1}{2} \text {Tr}(E_0 \left| \psi _0\right\rangle \left\langle \psi _0\right| ) + \frac{1}{2} \text {Tr}(E_1\left| \psi _1\right\rangle \left\langle \psi _1\right| ) = 1$.

### Components of the discrimination scheme and decompositions

We divide the implementation of our discrimination circuit into two distinct components: the discriminator and the measurement. The unknown unitary gates, representing the quantum channel to be identified, are inserted between these two parts. In practical experiments, we focus specifically on schemes that in theory achieve perfect discrimination between the identity operation and the $\text {RZ}(\phi )$ gate. To ensure that the condition previously discussed is met, we set the angle $\phi$ to $\pi / N$, where *N* denotes the total number of uses of the unknown gate in the circuit. This value is determined by the product of the width (the number of qubits used in parallel) and depth (the number of successive applications of the unknown gate).

After using the discriminator, the circuit must be in a maximally entangled state on *N* qubits in the form

15 $$\begin{aligned} \left| \psi \right\rangle = \frac{1}{\sqrt{2}} \left( \left| 0\right\rangle ^{\otimes N} + \alpha \left| 1\right\rangle ^{\otimes N} \right) . \end{aligned}$$

**Fig. 1:**
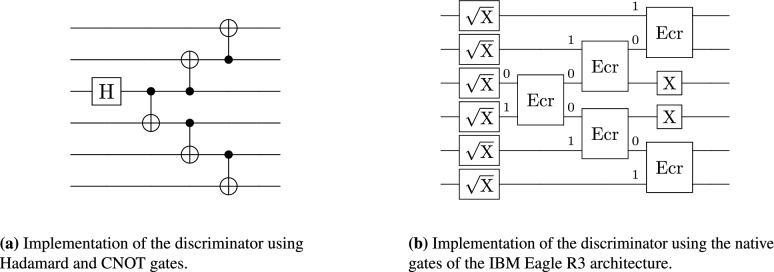
Schematic implementation of a six-qubit discriminator using different gate sets: (**a**) Hadamard and CNOT; (**b**) IBM Eagle R3 native basis (single-qubit $\sqrt{X}$ (SX) and two-qubit ECR).

To achieve this, we used a cascade of CNOT gates, as can be seen for six qubits in Fig. 1a. This discriminator is created based on the standard pattern that is commonly used to create the GHZ state^47^.

The experiments were carried out on IBM Brisbane, where the CNOT gate is not a native gate. To reduce the number of gates in the circuit, we prepared the discriminator specially designed for Eagle R3 architecture^48^. Then, we use ECR gates to create entanglement between qubits, while omitting CNOT gates unrolling during transpilation at the same time. The single-qubit gate $\sqrt{X}$ (SX) has matrix form $$\displaystyle \sqrt{X}=\tfrac{1}{2}\begin{pmatrix} 1+i & 1-i \\ 1-i & 1+i \end{pmatrix}$$. The first part of the discriminator consists of SX gates on all qubits and then a cascade of ECR gates of similar structure as the CNOT cascade in the first case. Unlike the discriminator based on CNOT gates, we also had to add several X gates to the end of the discriminator to get the desired quantum state. There is little to no pattern in the qubits on which the X gate has to be applied. Therefore, we prepared the discriminator for each number of qubits separately by hand. In Fig. 1b, we show the decomposition of six-qubit discriminator using ECR gates. As we could see, in this case the X gates are applied on second and third qubits.

**Fig. 2:**
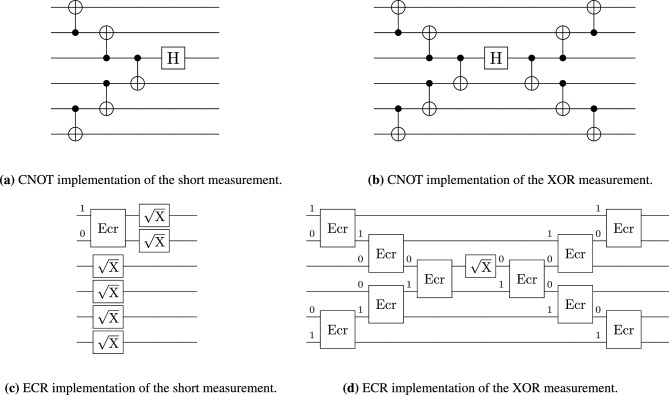
Schematic implementations of six-qubit measurement circuits across gate sets. Panels (**a**) and (**b**) depict the circuits for the short and XOR measurement schemes, respectively, implemented using the non-native $\text {H}$ and $\text {CNOT}$ basis. Panels (**c**) and (**d**) illustrate the circuits for the short and XOR measurement schemes, respectively, utilizing the native IBM Eagle R3 basis gates ($\sqrt{\text {X}}$ and ECR).

For measurement, two distinct circuit implementations were used. The first method, referred to as the short measurement approach, is characterized by a reduced circuit depth and overall gate count. This method produces the disjoint sets of possible measurement outcomes, one for each unitary channel. However, this advantage is offset by an increased susceptibility to bit-flip errors. This circuit was implemented using CNOT gates, presented in Fig. 2a, or ECR gates, as in Fig. 2c. For the CNOT-based implementation, the measurement outcomes exhibit a regular structure: the scheme for the identity channel consistently yields the all-zero bitstring, while for the unitary channel $\Phi _{\text {RZ}(\theta )}$ produces bitstrings that are zero in all positions except for a single qubit set to one. The second measurement approach, referred to as the XOR measurement, uses a much deeper circuit. However, an added benefit is that the result for the identity channel consists of all zeros, and the result for the unitary channel consists of all ones. This approach helps mitigate bit-flip errors: if the majority of measured bits are zero, the result is taken as all zeros. In the case of a six-qubit system, and in the absence of noise, the observed bitstrings are 000000 for identity and 111111 for the unitary channel $\Phi _{\text {RZ}(\theta )}$. The implementation of this circuit is shown in Fig. 2b.

In contrast, the ECR-based implementation returns a more complex distribution of output bitstrings, lacking the clear structure observed in the CNOT-based case. With this implementation, we get two disjoint subsets of all possible results, one subset for identity channel and the second subset for the unitary channel $\Phi _{\text {RZ}(\theta )}$. For example, on six-qubit system, we obtain the following two sets of bitstrings after measurement $\{001111, 010111, 101101, 110101, 100001, 100111, 011101, 110011, 000011, 111001, 011011, 111111, 001001, 000101, 010001, 101011\}$ for the identity channel and $\{110001, 001011, 011111, 100011, 001101, 110111, 000001, 010101, 000111, 101001, 111101, 010011, 101111, 111011, 011001, 100101\}$ for $\Phi _{\text {RZ}(\frac{\pi }{6})}$. Implementation of this ECR circuit could be seen in Fig. 2d.

The bitstring 001111 from the first set and 001101 from the second differ by only one bit, which may be problematic in a noisy environment. Even in this small example, processing these results is not straightforward, though this is compensated by the simplicity of the measurement circuit. In both measurement schemes, if it is not possible to determine which channel was applied during experiment, i.e., when the distance to both candidate channels is equal, then the output is assigned randomly via a coin flip. This approach ensures that each circuit run yields a definite outcome, thereby avoiding missing data points. Similarly, if the majority of bits have value one, we take this as the result of all of them.

#### Mapping on Eagle R3

- ECR gates are oriented, qubit order matters and simple swaps are constrained.
- For small widths *w*, hand-crafted ECR-native layouts avoid transpiler SWAP insertion.
- For larger *w*, either (i) size-specific hand layouts or (ii) transpiler-based layouts with SWAPs due to connectivity/orientation can be used.
- SWAPs increase depth and two-qubit gate count, impacting $p_{\text {succ}}$.

### Comparative analysis of transpilation

To evaluate the efficacy of different quantum circuit transpilation approaches, particularly concerning the choice between CNOT and ECR gates and the impact of manual qubit mapping, experiments were conducted on the 6-qubit and the 11-qubit systems. The objective is to compare the performance, measured by result accuracy, for circuits utilizing either the short measurement or the XOR-based measurement scheme, while varying the transpilation method. Each circuit was executed with 100,000 shots.

The selection of CNOT and ECR gates for this study is based on their fundamental role in creating entanglement, which is a crucial component of the quantum circuits being investigated. A key consideration in this comparison is the fundamental difference in how these gates are implemented on the hardware, particularly on the Eagle R3 architecture. On this system, the ECR gate is a native gate, meaning it can be executed directly by the hardware. In contrast, the CNOT gate is not native and must be unrolled into a sequence of native gates. Theoretically, a single CNOT gate requires one ECR gate plus additional single-qubit gates, already leading to a deeper circuit^49^. Furthermore, the ECR gates on the Eagle R3 are oriented, meaning the qubit order matters and simple swaps are constrained. This necessitates careful mapping. For circuits with a small number of qubits, hand-crafted ECR-native layouts can be designed to avoid the insertion of additional SWAP gates by the transpiler. However, for larger circuits, either size-specific manual layouts or transpiler-based layouts are used. The latter often results in the automatic insertion of SWAP gates to accommodate qubit connectivity and gate orientation. The presence of these SWAP gates, which are themselves implemented as a sequence of native gates, significantly increases the circuit depth and the two-qubit gate count. This increased depth leads to a higher accumulation of errors, which directly impacts the result accuracy^50^. Detail characteristics for different transpilation and measurement strategies for both 6-qubit and 11-qubit can be seen in Table 1.

#### 6-Qubit system evaluation

For the 6-qubit configuration in a pure parallel discrimination scheme, four distinct transpilation strategies were evaluated using the short measurement and XOR-based measurement protocols:

1. **CNOT + Transpiler:** Circuit implemented using CNOT basis gates, processed by the Qiskit transpiler with optimization level 3. Short accuracy: $88.8\%$; XOR accuracy: $86.4\%$.
2. **ECR + Transpiler:** Circuit implemented using ECR basis gates, processed by the Qiskit transpiler with optimization level 3. Short accuracy: $83.8\%$; XOR accuracy: $90.0\%$.
3. **ECR + Transpiler + Fixed Mapping:** Circuit implemented using ECR basis gates, processed by the Qiskit transpiler with optimization level 3 and a predetermined, fixed mapping of logical qubits to physical qubits. Short accuracy: $84.4\%$, XOR accuracy: $85.3\%$.
4. **ECR + Fixed Mapping (No Opt.):** Circuit implemented using ECR gates, utilizing a fixed logical-to-physical qubit mapping, bypassing subsequent transpiler optimization passes. Short accuracy: $83.3\%$, XOR accuracy: $85.6\%$.

The experimental results indicate that for this system size, applying a fixed qubit mapping does not yield a discernible improvement in accuracy compared to relying solely on the transpiler’s default ECR implementation. This observation is potentially attributable to the limited circuit depth and complexity inherent in six-qubit systems, where the transpiler’s optimization might already find near-optimal solutions without explicit mapping constraints.

#### 11-Qubit system evaluation

A subsequent set of experiments was performed using the 11-qubit system. Five transpilation strategies were assessed for each measurement type:

1. **CNOT + Transpiler:** Circuit using CNOT basis gates, transpiled with optimization level 3. Short accuracy: $43.3\%$, XOR accuracy: $48.5\%$.
2. **ECR + Transpiler:** Circuit using ECR basis gates, transpiled with optimization level 3. Short accuracy: $55.0\%$, XOR accuracy: $54.5\%$.
3. **ECR (Topology-Aware) + Transpiler:** Circuit initially designed considering device connectivity using ECR gates, then transpiled with optimization level 3. Short accuracy: $36.1\%$, XOR accuracy: $47.2\%$.
4. **ECR (Topology-Aware) + Transpiler + Fixed Mapping:** Topology-aware ECR circuit, transpiled with optimization level 3 and a fixed logical-to-physical qubit mapping. Short accuracy: $32.0\%$, XOR accuracy: $71.5\%$.
5. **ECR (Topology-Aware) + Fixed Mapping (No Opt.):** Topology-aware ECR circuit with fixed mapping, bypassing subsequent transpiler optimization. Short accuracy: $33.4\%$, XOR accuracy: $71.8\%$.

The results for the 11-qubit system demonstrate a general performance advantage for ECR-based implementations over CNOT-based ones for both measurement protocols. For the short measurement scheme, the standard ECR implementation processed by the transpiler (Method 2) yields the highest accuracy ($55.0\%$). However, a more pronounced effect is observed for the XOR measurement protocol. Employing a topology-aware circuit design combined with fixed qubit mapping (Methods 4 and 5) results in a significant accuracy improvement, achieving approximately 71.5–71.8%, nearly a $20\%$ absolute increase compared to the standard CNOT transpiled approach ($48.5\%$).

To assess the effect of *Transpilation Strategy* and *Measurement Scheme* on accuracy in the hardware experiments summarized in the circuit-statistics tables, we performed a two-way ANOVA without replication ^51^. For the 6-qubit system, neither factor was statistically significant at $\alpha =0.05$ (*Transpilation Strategy*: $F(3,3)=0.745$, $p=0.593$; *Measurement Scheme*: $F(1,3)=0.966$, $p=0.398$). For the 11-qubit system *Transpilation Strategy* remained non-significant ($F(4,4)=0.327$, $p=0.848$), while *Measurement Scheme* showed a marginal, non-significant trend ($F(1,4)=4.914$, $p=0.091$). The modest sample size for the 11-qubit analysis limits statistical power, despite clear descriptive differences in some configurations.

The current experimental design, which utilized only a single accuracy result for each Transpilation Strategy and Measurement Scheme combination, precluded the testing of interaction effects between these categories for both the 6-qubit and 11-qubit systems, investigating these likely but untested relationships will be essential for future, more robust research. It is also worth observing that the substantial accuracy gain observed for the 11-qubit XOR measurement using hardware-aware design and fixed mapping highlights the potential benefits of tailoring circuits to specific device characteristics. However, this approach presents a significant practical challenge: it currently necessitates manual, device-specific, and qubit-count-specific circuit construction and mapping. This process lacks straightforward algorithmic automation and requires considerable expert intervention for each target configuration, limiting its scalability and general applicability.

**Table 1:** Circuit complexity metrics for the 6-qubit system presented in Table (a) and for the 11-qubit system presented in Table (b) across transpilation strategies. The table details the circuit *Depth*, two-qubit gate count (ECR), and single-qubit gate counts (RZ, $\sqrt{\text {X}}$/SX, X, ID/$\text {RZ}(\phi )$) after transpilation to the native basis of the IBM Eagle architecture, comparing different initial circuits and measurement schemes. For the 11-qubit system the entries “ECR + Transpiler (ID)” and “ECR + Transpiler ($\text {RZ}(\phi )$)” reflect special cases where the measurement or state discrimination was tuned for the Identity (ID) and $\text {RZ}(\phi )$ channel, respectively. This specialized setting directly influenced the transpiler’s output, leading to the distinct final circuit properties observed in these rows.

(a) Circuit statistics for 6-qubits experiments							
Transpilation strategy	Measurement	Depth	ECR	RZ	$\sqrt{\text {X}}$	X	ID/$\text {RZ}(\phi )$
CNOT + Transpiler	Short	35	10	53	30	5	6
CNOT + Transpiler	XOR	44	14	62	36	9	6
ECR + Transpiler	Short	20	6	18	19	1	6
ECR + Transpiler	XOR	40	14	59	34	2	6
ECR + Transpiler + Fixed Mapping	Short	8	6	0	12	2	6
ECR + Transpiler + Fixed Mapping	XOR	21	14	7	12	2	6
ECR + Fixed Mapping (No Opt.)	Short	8	6	0	12	2	6
ECR + Fixed Mapping (No Opt.)	XOR	13	15	0	7	2	6

(b) Circuit statistics for 11-qubits experiments.							
Transpilation strategy	Measurement	Depth	ECR	RZ	$\sqrt{\text {X}}$	X	ID/$\text {RZ}(\phi )$
CNOT + Transpiler	Short	63	20	99	58	10	11
CNOT + Transpiler	XOR	65	29	108	52	18	11
ECR + Transpiler	Short	31	11	30	32	2	11
ECR + Transpiler (ID)	XOR	81	32	108	65	3	11
ECR + Transpiler ($\text {RZ}(\phi )$)	XOR	85	35	115	66	4	11
ECR (Topology-Aware) + Transpiler	Short	26	11	23	30	2	11
ECR (Topology-Aware) + Transpiler	XOR	70	29	108	62	6	11
ECR (Topology-Aware) + Transpiler + Fixed Mapping	Short	11	11	0	22	2	11
ECR (Topology-Aware) + Transpiler + Fixed Mapping	XOR	30	29	7	17	2	11
ECR (Topology-Aware) + Fixed Mapping (No Opt.)	Short	11	11	0	22	2	11
ECR (Topology-Aware) + Fixed Mapping (No Opt.)	XOR	22	30	0	12	2	11

### Preliminary results

**Fig. 3:**
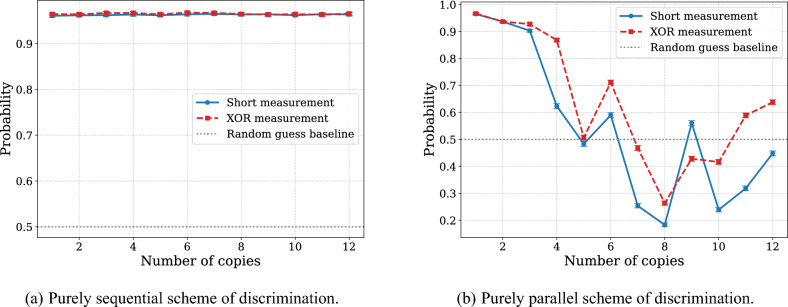
Probability of successful discrimination between the identity operation and the $\text {RZ}(\pi / N)$ gate, where *N* is the number of copies of unknown unitary. The dashed red line corresponds to the XOR-based measurement strategy, while the solid blue line represents the short measurement scheme. Figure (**a**) illustrates the performance of the purely sequential scheme. In Figure (**a**), the probability is approximately 0.965. Figure (**b**) corresponds to the purely parallel scheme. Each circuit was executed with 10,000 shot. Measurement outcomes that could not be unambiguously associated with either gate were randomly assigned to one of the two possible answers.

From the initial experiments involving purely sequential and purely parallel discrimination schemes (see Fig. 3), we observe that the use of entangling gates on real quantum devices introduces a greater error overhead than the decoherence effects arising from increased gate depth in sequential protocols.

**Fig. 4:**
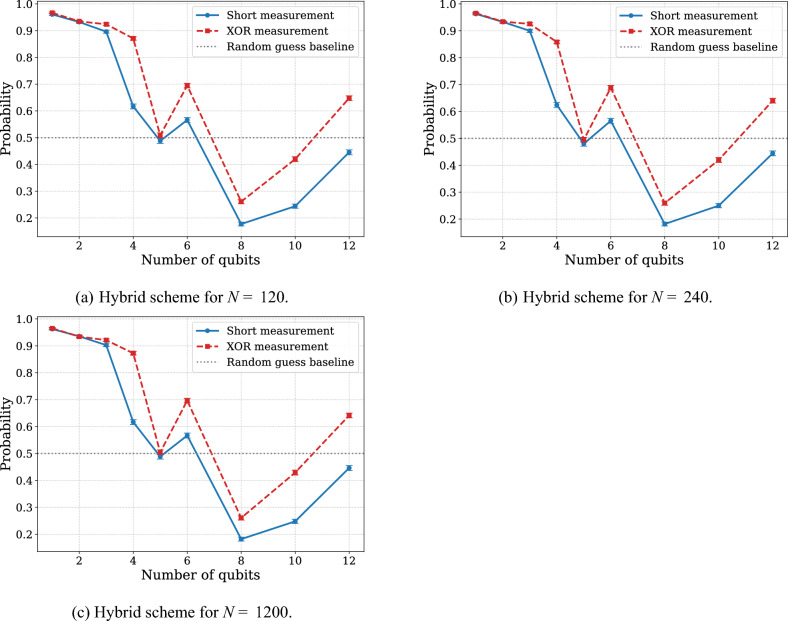
Probability of successful discrimination between the identity operation and the RZ($\pi /N$) gate, where *N* is number of copies of unknown unitary, plotted as a function of the width of the hybrid rectangular scheme. The dashed red line corresponds to the XOR-based measurement strategy, while the solid blue line represents the short measurement scheme. Figure (**a**) illustrates the performance of hybrid scheme with 120 copies in total, Figure (**b**) corresponds to hybrid scheme with 240 copies in total, and Figure (**c**) corresponds to hybrid scheme with 1200 copies in total. Each circuit was executed with 10,000 shots. Measurement outcomes that could not be unambiguously associated with either gate were randomly assigned to one of the two possible answers.

To further investigate this observation, we conducted a series of tests using hybrid rectangular (sequentially-parallel) schemes with a fixed number of unknown gate applications (see Fig. 4). The results consistently show a significant increase in error rates as more entangling gates are introduced. This trend reinforces the conclusion that the primary source of performance degradation in current devices stems from the imperfections of multi-qubit gate operations rather than decoherence from circuit depth alone. Another notable observation is that different measurement schemes have a visible but not dominant impact on the overall error rate, further suggesting that the circuit width and the number of entangled qubits are the primary contributors to performance degradation. This reinforces the conclusion that the main source of error arises from the entanglement rather than from the specifics of the measurement strategy.

### Anomalous behavior discussion

During the experiments, we observed a specific issue with the IBM Quantum platform: measurements in experiments involving the entanglement of a certain number, typically five or more, qubits exhibited random bit-flip errors. This type of error is evident in Figs. 3 and 4 and appears to affect all qubits simultaneously, independent of the measurement strategy. Given that random guessing yields a baseline success probability of 0.5, device noise would normally drive the measured success rates toward this value. Instead, we observed something like inversion of outcomes, effectively corresponding to a swap of the expected answer sets. These anomalies can be reversed by interchanging the target labels, which restores internal consistency in the data. However, this procedure is purely for illustration, as the underlying cause of the bit-flip behavior remains unidentified and such swapping does not constitute a valid mitigation strategy. To ensure that the issue was not caused by errors in our own implementation, we performed several verification steps. First, multiple experimental results from different dates were analyzed. Second, the final transpiled circuits were simulated using a noiseless simulator to confirm the expected theoretical behavior. Third, we applied M3 error mitigation techniques^52^, which had no measurable effect on the observed phenomena. Given that the observed errors persist across all experiments, regardless of the RZ gate angle or the measurement strategy employed, we hypothesize the presence of a systematic hardware or software artifact specific to the IBM Quantum platform, particularly the Brisbane quantum device. Further investigation and hypothesis-driven testing are required to understand this behavior.

## Experiment 2—Discrimination of unitary channels on IBM Brisbane with processing

In this section, we will present the second example of discrimination task with a different pair of unitary channels that require intermediate processing between applications to be perfectly distinguishable and run it on IBM Brisbane.

### Methodology

In the second experiment, we take the following unitary channels to discriminate $\Phi _U$ for $U = \sqrt{X}\textrm{RZ}(\frac{-\pi }{2N})\sqrt{X}$ and $\Phi _V$ for $V = \sqrt{X}\textrm{RZ}(\frac{\pi }{2N})\sqrt{X}$, where $$\sqrt{X} = \frac{1}{2} \begin{pmatrix} 1+i & 1-i\\ 1-i & 1+i \end{pmatrix}$$. It is easy to check that the condition $\theta (V^\dagger U) = \frac{\pi }{N}$ is satisfied. It implies that for *N*-shot discrimination scenario, we will achieve perfect discrimination. Let us fix $N = wd$, where $w,d \in \mathbb {N}$. In this setup, instead of mid-processing $X_i = (V^\dagger )^{\otimes w}$ we use hardware-friendly processing $X_i = X^{\otimes w}$. For convenience we add also pre-processing unitary operation $X_0 = (X\sqrt{X})^{\otimes w}$ and post-processing operation $X_d = (\sqrt{X}X)^{\otimes w}$. Combining processing operators with the black-box $\Phi _U$ we get the following unitary operation

16 $$\begin{aligned} U_* = X_dU^{\otimes w}X_{d-1}\cdots X_1U^{\otimes w}X_0 = \left( \textrm{RZ}(\frac{-\pi }{2N})^d\right) ^{\otimes w}. \end{aligned}$$

Similarly, for $\Phi _V$ the combined unitary circuit equals $V_* = \left( \textrm{RZ}(\frac{\pi }{2N})^d\right) ^{\otimes w}$. Note that $\theta (\left( \textrm{RZ}(\frac{-\pi }{2N})^d\right) ^{\otimes w}\left( \textrm{RZ}(\frac{-\pi }{2N})^d\right) ^{\otimes w}) = wd\theta (\textrm{RZ}(\frac{-\pi }{N})) = N\frac{\pi }{N} = \pi$, so in theory, each shape *w*, *d* gives perfect discrimination. As $X, \sqrt{X}, \textrm{RZ}$ are native gates in IBM Quantum Brisbane this part of the circuit is implemented exactly as stated. To define the discriminator $\left| \psi \right\rangle$, we solve

17 $$\begin{aligned} \left\langle \psi \right| \left( \textrm{RZ}(\frac{-\pi }{N})^d\right) ^{\otimes w}\left| \psi \right\rangle = 0. \end{aligned}$$

We find that the input state can be taken as the GHZ state $\left| \psi \right\rangle = \frac{1}{\sqrt{2}}(\left| 0\ldots 0\right\rangle + \left| 1\ldots 1\right\rangle )$, which can be implemented by Hadamard gate followed by cascade of control-X gates. We let the IBM transpiler to optimize the input state circuit with the optimization level set to 3.

We distinguish two situations depending on the value of $\Phi$. If $\Phi = \Phi _U$, then the input state $\left| \psi \right\rangle$ evolved under $U_*$ is equal to $\left| \psi _{-i}\right\rangle = \frac{1}{\sqrt{2}}(\left| 0\ldots 0\right\rangle - i \left| 1\ldots 1\right\rangle )$. For $\Phi = \Phi _V$ we get after the evolution $V_*$ that $\left| \psi _{i}\right\rangle = \frac{1}{\sqrt{2}}(\left| 0\ldots 0\right\rangle + i \left| 1\ldots 1\right\rangle )$. At the measurement stage we implement $\textrm{RZ}(\pi /2)$ on the first qubit followed by parallel application of Hadamard gates $H^{\otimes w}$. Each qubit is then measured in the *Z*-basis. We let the IBM transpiler to optimize the measurement circuit with the optimization level set to 3. At the measurement stage, after applying $\textrm{RZ}(\pi /2)$ and $H^{\otimes w}$ we get

18 $$\begin{aligned} H^{\otimes w}(\textrm{RZ}(\pi /2) \otimes \mathrm{1\hspace{-0.9mm}l})\left| \psi _{\mp i}\right\rangle = \left| +\cdots +\right\rangle \pm \left| -\cdots -\right\rangle , \end{aligned}$$

where we used the notation of plus/minus states $\left| +\right\rangle = H\left| 0\right\rangle , \left| -\right\rangle = H\left| 1\right\rangle$. Observe that if $(b_1,\ldots ,b_w)$ is a bit string we received from measuring $\left| +\cdots +\right\rangle \pm \left| -\cdots -\right\rangle$, then in theory, $\Phi = \Phi _U$ if and only if $b_1 \oplus \cdots \oplus b_w \equiv 0$, where $\oplus$ denotes XOR operation between bits. Similarly, $\Phi = \Phi _V$ if and only if $b_1 \oplus \cdots \oplus b_w \equiv 1$.

### Preliminary results

**Fig. 5:**
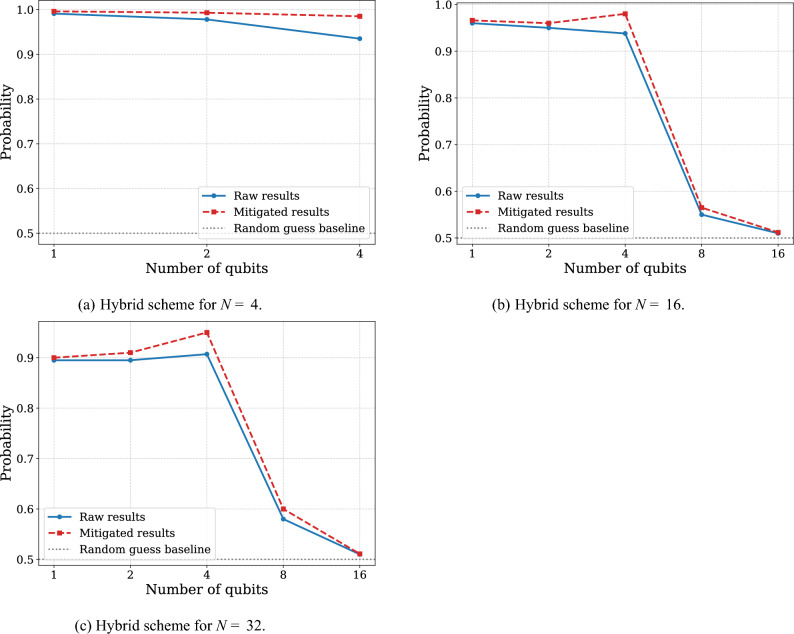
Probability of successful discrimination between the unitary operator $U = \sqrt{X} \textrm{RZ}(\frac{-\pi }{2N}) \sqrt{X}$ and $V = \sqrt{X} \textrm{RZ}(\frac{\pi }{2N}) \sqrt{X}$, where *N* is number of copies of unknown unitary, plotted as a function of the width of hybrid rectangular scheme, using the short measurement. The blue line corresponds to the results obtained directly from IBM Brisbane, while the red line represents the results after error mitigation using MThree package^52,53^. Figure (**a**) illustrates the performance of hybrid schemes with 4 copies in total, Figure (**b**) corresponds to hybrid schemes with 16 copies in total, and Figure (**c**) corresponds to hybrid schemes with 32 copies in total. Each circuit was executed with 10,000 shots.

**Fig. 6:**
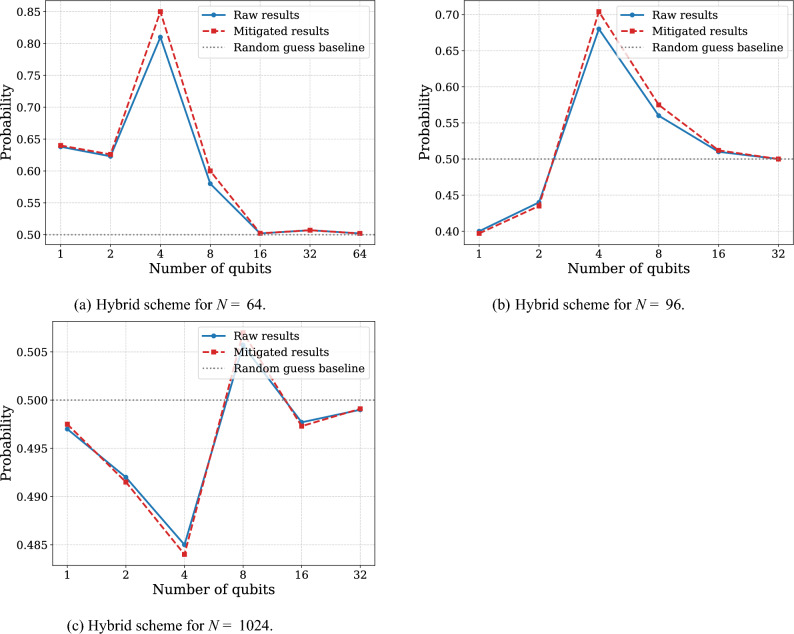
Probability of successful discrimination between the unitary operator $U = \sqrt{X} \textrm{RZ}(\frac{-\pi }{2N}) \sqrt{X}$ and $V = \sqrt{X} \textrm{RZ}(\frac{\pi }{2N}) \sqrt{X}$, where *N* is number of copies of unknown unitary, plotted as a function of the width of hybrid rectangular scheme, using the short measurement. The blue line corresponds to the results obtained directly from IBM Brisbane, while the red line represents the results after error mitigation using MThree package^52,53^. Figure (**a**) illustrates the performance of hybrid schemes with 64 copies in total, Figure (**b**) corresponds to hybrid schemes with 96 copies in total, and Figure (**c**) corresponds to hybrid schemes with 1024 copies in total. Each circuit was executed with 10,000 shots.

From relatively small number of copies ($N=4,16,32$) of unitary channels to be discriminated, the experiments involving purely sequential schemes are preferable (see Fig. 5). We observe that the use of entangling gates on real quantum devices introduces a higher error overhead than the decoherence effects arising from increased gate depth in sequential protocols. For an increasing number of copies ($N=64, 96, 1024$) of unitary channels, we can observe the advantage of usage sequentially-paralleled schemes to achieve more precise results (see Fig. 6). The results consistently show that after some threshold of gate composition, the existing decoherence or accumulative calibration imperfections gives higher error rates than the error rates used to create an entangled state.

### Discussion

In the experiment with $N=1024$ copies of black-box, we obtained effectively random results for all schemes considered (see Fig. 6c). The sequential scheme required excessive gate composition, while the parallel scheme required preparing a GHZ state that was too large. Both circuits introduced too many errors to yield any notable results. The final question that we can ask is if we can do better than that by exploring *suboptimal* circuits. A simple idea goes as follows. We perform independent sequential experiments on each of the *w* qubits. For each, we compose *d* quantum circuits and do independent measurements that indicate which black-box is preferable. As $\theta (V^\dagger U) = \frac{\pi }{N}$ for each qubit, we get the maximum angle spread of $\theta = \frac{\pi }{N}d = \frac{\pi }{w} < \pi$. Hence, the protocol is suboptimal. The probability of successful discrimination can be boosted by using majority voting on the results collected from the quantum computer. Let us assume that we collected *k* times label 0 indicating $\Phi _U$ and $w-k$ times label 1 standing for $\Phi _V$. If $k > w-k$ we guess that $\Phi = \Phi _U$ (and for $k < w-k$ we guess $\Phi = \Phi _V$). In the case $k=w-k$, we make a random guess.

We applied the following suboptimal procedure for $N=1024$. We used $w=32$ qubits, and on each qubit we applied the operation $\Phi$ sequentially $d=32$ times. According to Fig. 5, we should expect around $90\%$ accuracy for each qubit. In combination, we obtained $p_{\textrm{succ}} = 0.56765$, which is better than any optimal scheme. Similarly, we conducted the experiment for $N = 96$ and $w=3, d=32$ resulting in $p_{\textrm{succ}} = 0.74685$, which is a better result than indicated by Fig. 6b.

Can we say that suboptimal circuits are favorable for unitary channel discrimination? The answer is not straightforward. For example, for $N = 64$, the best result from Fig. 6a is slightly better than the suboptimal procedure presented above, even in the absence of noise, with $p_{\textrm{succ}} = 0.85295$. Interestingly, no analogous bit-flip anomalies were observed in these results compared to Experiment 1 (page 8), even though they employ comparable qubit counts and circuit depths. This may suggest that updates to the IBM Quantum execution pipeline or internal compilation procedures now perform additional low-level optimizations or calibration corrections before execution on hardware, which could explain the collective measurement flips seen in the simpler example. However, since these mechanisms are not publicly documented, this explanation remains speculative.

## Noise modeling and ablation analysis

### Theoretical noise model

In this section we analyze the effect of symmetric depolarizing noise on the circuit presented in Experiment 1 for both optimal and suboptimal discrimination strategies. Let $U_\pm = \textrm{RZ}(\pm \pi /(2N))$ be two unitary operations to distinguish, where *N* is the number of copies given. We assume that $N = r w d$, where *r* is the number of independent circuits prepared, *w* is the number of qubits used per circuit, and *d* is the number of compositions of $U_\pm$ in each register. As a universal gate set, we choose single qubit operations and CNOT gates. Finally, the depolarizing noise is modeled as follows. Let $\Omega _\epsilon$ be a parametrized qubit depolarizing noise given by the equation $\Omega _\epsilon (\rho ) = (1-\epsilon ) \rho + \epsilon \rho _*$, with the noise ratio $\epsilon \in [0,1]$ and $\rho _*$ indicating maximally mixed state. Each single qubit gate *U* is affected by $\Omega _\epsilon$, which we write as $\Omega _\epsilon (U\rho U^\dagger )$. The CNOT gate is affected by two independent noise sources acting on both qubits, $(\Omega _\epsilon \otimes \Omega _\epsilon )(CNOT \rho CNOT^\dagger )$. To implement suboptimal strategies, we use majority voting for *r* independent experiments. Hence, we will consider only odd values of *r*. If $r=1$, we have the optimal strategy.

To implement the input state, we use single Hadamard gate and CNOT ladder containing $w-1$ two-qubit operations. Hence, for the noiseless circuit ($\epsilon =0$) we get a generalized GHZ state $\frac{1}{\sqrt{2}}(\left| 0\cdots 0\right\rangle + \left| 1\cdots 1\right\rangle )$. We further implement *wd* unitary $U_\pm$ gates as $(U_{\pm }^d)^{\otimes w}$ and obtain the state $\frac{1}{\sqrt{2}}(\left| 0\cdots 0\right\rangle + e^{\pm (\pi wd)/(2N)i}\left| 1\cdots 1\right\rangle )$. The measurement is performed using the short-measurement circuit, where on the first qubit we put the gate $$\frac{1}{\sqrt{2}}\begin{pmatrix} 1 & i\\ 1 & -i \end{pmatrix}$$ and on the remaining $w-1$ qubits we put Hadamard gates. As a result, the state to measure is of the form $\frac{1}{\sqrt{2}}(\left| +\cdots +\right\rangle + ie^{\pm (\pi wd)/(2N)i}\left| -\cdots -\right\rangle ).$ For any bitstring $b_1,\ldots ,b_w$ satisfying $b_1 \oplus \cdots \oplus b_w = 0$, where $\oplus$ denotes XOR operation between bits, we get the probability $P(b_1,\ldots ,b_w) = \frac{1}{2^{w+1}}\left| 1+i e^{\pm (\pi wd)/(2N)i}\right| ^2 = \frac{1}{2^{w}}\left( 1 \mp \sin \left( \frac{\pi }{2r}\right) \right) .$ Therefore, each bitstring with the property $b_1 \oplus \cdots \oplus b_w = 0$ will be connected with the guess $U_-$ and $b_1 \oplus \cdots \oplus b_w = 1$ with $U_+$. In total, the success probability for correct discrimination in a single noiseless experiment reads $p_s = \frac{1}{2}(1+\sin \left( \frac{\pi }{2r}\right) )$.

In the presence of the noise $\epsilon > 0$ there is nonzero probability that at some point of the circuit, single qubit will decohere to $\rho _*$. If a single event like this happen, then the full coherence of the quantum state is lost. The information about $U_\pm$ is held in the phase of the GHZ state. Therefore, if at least one error occurs, the information about $U_\pm$ is lost and the probability of correct discrimination drops to 1/2. Let us count the probability that there is no errors. Each error can occur with the same probability $\epsilon$ independently, so we need to count the number of places where the error can happen. To create the GHZ state, we use a single Hadamard gate and $w-1$ CNOT gates. In total, there are $1+2(w-1)$ possible positions where an error can occur. Then, we implement $U_\pm$, which adds *d* additional error positions per qubit, giving *dw* in total, where *w* is the circuit width. Finally, at the measurement stage we implement Hadamard gate for each qubit resulting in *w* error positions. To sum up, there are $1+2(w-1)+wd+w = wd + 3w - 1$ error positions. The probability that there is no error is then $(1-\epsilon )^{wd+3w-1}$. The success probability for a single experiment in the presence of noise is then given by

19 $$\begin{aligned} p_s(\epsilon ) = (1-\epsilon )^{wd+3w-1}p_s + (1- (1-\epsilon )^{wd+3w-1})\frac{1}{2} = \frac{1}{2}+\frac{1}{2} (1-\epsilon )^{wd+3w-1}\sin \left( \frac{\pi }{2r}\right) . \end{aligned}$$

Finally, if we add the majority vote procedure for *r* independent experiments, the total probability of success in the presence of noise $\Omega _\epsilon$ reads as follows

20 $$\begin{aligned} P_{r,w,d}(\epsilon ) = \sum _{k = \lceil r/2\rceil }^r \left( {\begin{array}{c}r\\ k\end{array}}\right) p_s(\epsilon )^k(1-p_s(\epsilon ))^{r-k}. \end{aligned}$$

If $r=1$ and $\epsilon =0$ we see that $P_{1,w,d}(0)=1$ as intended for any shape (*w*, *d*). However, in the presence of noise, we see that from all optimal strategies $(r=1)$, the formula $wd+3w-1=N+3w-1$ is minimized for circuits with fewer qubits. Therefore, the circuits with a usage of a single qubit are preferable. They achieve a probability of success $P_{1,1,N}(\epsilon ) = \frac{1}{2}+\frac{1}{2} (1-\epsilon )^{N+2}$. The comparison with suboptimal strategies is more complex. As suboptimal strategies ($r > 1$) involve usage of majority voting they naturally constitute error correction method. Therefore, they could be preferable when the noise level exceeds some level for a given problem size. Indeed, let us take as an example $N=150$ and compute $P_{1,1,150}(0.01) \simeq 0.6085$ and $P_{3,1,50}(0.01) \simeq 0.7158$. Three times repetition provides advantage for $\epsilon =0.01$ even if for noiseless system we have $P_{1,1,150}(0) = 1$ and $P_{3,1,50}(0)= 0.84375$.

In conclusion, for a given quantum device with fixed error rate, large enough discrimination problems will benefit from utilizing suboptimal procedures that are more prone to errors.

### Ablation study

To quantify the effect of different noise sources in Experiment 2, we performed an ablation study with the Aer Simulator using parameters calibrated to IBM Brisbane. Instead of a single model, we constructed configurations that enable only specific channels:

- 1q only: depolarization on single-qubit gates,
- 2q only: depolarization on two-qubit gates,
- T$_1$/T$_2$ only: thermal relaxation and dephasing,
- Readout only: asymmetric measurement errors,
- Full rebuilt: manual composition of average depolarization, $T_1/T_2$, and readout noise,
- Full backend: calibration-based model from NoiseModel.from_backend.

The calibration data used for noise modeling were derived from the real IBM Brisbane device around the time of the experiments. The average single-qubit depolarizing error was set to $1.104\times 10^{-3}$, and the two-qubit (ECR) gate error to $1.946\times 10^{-2}$. The median relaxation times were taken as $T_1 = 210~\mu \text {s}$ and $T_2 = 155~\mu \text {s}$, with corresponding gate durations of $35~\text {ns}$ for single-qubit gates and $248~\text {ns}$ for two-qubit gates. The readout errors were modeled with asymmetric probabilities of $p_{01}=2.5\times 10^{-2}$ and $p_{10}=2.0\times 10^{-2}$.

**Fig. 7:**
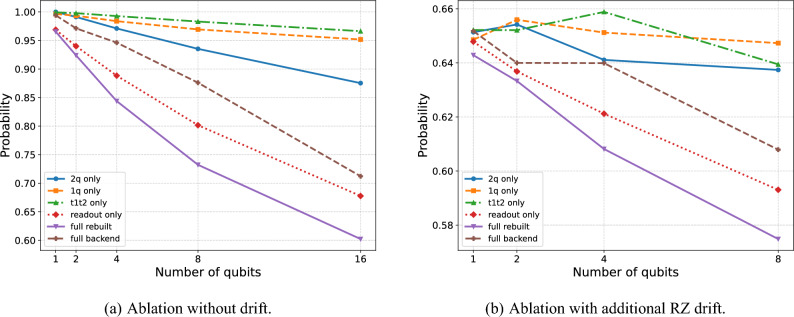
Probability of successful discrimination between $U=\sqrt{X},\textrm{RZ}(-\pi /128),\sqrt{X}$ and $V=\sqrt{X},\textrm{RZ}(\pi /128),\sqrt{X}$ for $N=64$ copies, plotted as a function of circuit width *w* (number of qubits). The dashed brown line corresponds to the full backend noise model of IBM Brisbane provided by Qiskit, while the solid purple line denotes the full rebuilt model that combines depolarization, thermal relaxation, and readout errors. The remaining curves represent ablation configurations with only single-qubit noise (dashed orange), two-qubit noise (solid blue), thermal relaxation (dash-dotted green), or readout noise (dotted red). The results confirm that two-qubit and readout noise are the dominant contributors to performance degradation. Figure (**a**) shows ablation results without coherent drift, whereas Figure (**b**) includes an added systematic phase offset $\theta \mapsto \theta +\pi /160$ on each $\textrm{RZ}$ gate. Each circuit was executed with 10,000 shots.

The results for $N=64$ and widths $w\in \{1,2,4,8,16\}$ are shown in Fig. 7. The data confirm that two-qubit noise is the dominant source of degradation, while single-qubit errors and relaxation processes have a noticeably weaker but still increasing impact as the depth grows. Readout errors alone contribute greatly to the reduction in performance. However, this is likely an overestimation because in our noise model we apply uniform readout asymmetries rather than the full per-qubit readout map. The *full rebuilt* model consistently yields lower success probabilities than the *full backend*, as the reconstruction uses averaged $T_1/T_2$ values and applies depolarization and dephasing independently at each gate and also already mentioned readout errors. This slightly overestimates the combined effect of noise and neglects favorable device inhomogeneities, making the rebuilt model more pessimistic.

To further bridge the gap between simulations and hardware runs, we introduced a small coherent drift (biased gate calibration error) by replacing every $\textrm{RZ}(\theta )$ with $\textrm{RZ}(\theta +\pi /160)$. This modification yields the curves shown in Fig. 7b, which more closely reproduce the relative trends observed on real hardware (Fig. 6a), although the agreement is not exact.

## Conclusion

In this work, we have studied the discrimination of two quantum unitary channels and benchmarked various schemes for perfect discrimination between them. The benchmarks were performed using the IBM Brisbane quantum device. All figures indicate the shot count (10,000 shots per circuit). Where multiple hardware runs were available, we aggregated by computing $p_{\textrm{succ}}$ for each run and comparing across configurations as above. For noise-model simulations, we used the same success metric and classification rule as the hardware runs to ensure comparability.

As the first example, we chose the discrimination task between the identity operation and the RZ$(\phi )$ gate with no processing between them. To optimize circuit performance for this discrimination task, we also evaluated different quantum circuit transpilation approaches on 6- and 11-qubit subsystems, comparing the circuit implementation with CNOT and ECR gates and the impact of manual qubit mapping. Although fixed qubit mapping did not significantly improve accuracy in the smaller 6-qubit system, topology-aware circuit design combined with fixed mapping yielded substantial gains on the 11-qubit system when using XOR-based measurements, thereby underscoring the importance of hardware-aware optimization for larger circuits. In this experiment, we find the trend that deeper circuit architectures, which minimize entanglement overhead while preserving discrimination power, are significantly more resilient to hardware noise (see Fig. 3). Our results suggest that algorithm designers should prioritize circuit depth over width whenever possible. In the second example, we considered the discrimination between the unitaries $U = \sqrt{X},\textrm{RZ}(-\pi /2N),\sqrt{X}$ and $V = \sqrt{X},\textrm{RZ}(\pi /2N),\sqrt{X}$, where the processing consists of compositions of *X* and $\sqrt{X}$ gates. There, we have observed the advantage of using sequentially-paralleled schemes to achieve more precise results. The results consistently show that beyond a certain threshold of gate composition, decoherence and cumulative calibration imperfections produce higher error rates than those required to generate an entangled state.

Our calibrated noise simulations identify two-qubit gate and readout errors as primary performance limitations. Consequently, for large-scale problems such as $N=1024$, theoretically optimal schemes failed, producing random outcomes. In contrast, a suboptimal approach using majority voting proved more effective in some cases in accordance with the theoretical results. A final observation concerns the bit-flip anomaly reported in Experiment 1 (see Figs. 3 and 4), where correlated flips were observed across all measured qubits for certain qubit counts. In contrast, no such behavior was observed in Experiment 2 (see Figs. 5 and 6), despite its comparable width and depth combinations. This discrepancy may indicate that the IBM Quantum execution stack performs undisclosed optimizations or internal circuit simplifications for certain circuit classes, potentially suppressing these systematic artifacts in more complex layouts. However, without public access to the complete compilation and calibration pipeline, this hypothesis remains highly speculative. More research is needed to determine whether these effects arise from backend-level preprocessing, device-specific behavior, or a combination of both.

Circuit geometries beyond square layouts may offer a more accurate reflection of the capabilities of the device. These findings can be applied to various black-box tasks with many copies, such as quantum phase estimation ^54^. In that case, discrimination schemes that are theoretically suboptimal achieve good experimental performance ^55^. While comparing unitary channels leads to expected consistent and comparable results, the task of general quantum channel discrimination introduces complexity as its necessary implementation via the Stinespring representation requires adding ancillary systems and SWAP gates to enable entanglement. This operational overhead inherently subjects the experimental outcomes to a greater degree of error. Experimental studies of the discrimination of general quantum channels remain an open direction for future research.

## Data Availability

The data that support the findings of this study are openly available in Github repository at https://github.com/Dotnester/experimental_study_of_multiple_shot_channel_discrimination and on Zenodo^56^.
